# The Ploughman

**Published:** 1874-11

**Authors:** 


### THE PLOUGHMAN

Is considered the embodiment of general health and youthful vigor, arising from three considerations:

1st, He is breathing pure out-door air from morning until night.

2d, He performs his work with steady moderation.

3d, He exercises a great deal.

Whatever involves these three things will maintain health, and would cure three-fourths of our ordinary maladies; expressed in half a dozen words, "Moderate exercise in the open air."

It is a good summer's work to cultivate twenty acres of corn; but in doing so, a man must be pretty busy, for he will have to travel on foot nearly or quite one thousand miles. Almost all sedentary persons do themselves great harm in exercising. The idea is, that if exercise does good, the more violent it is, the greater are its advantages; hence, a clergyman saws his wood as if he had to get through a cord in half an hour, timing himself to a stick a minute, when he ought to give it fifteen. Result, gives out in thirty minutes, gets overheated, cools off too quickly, takes cold, feels sick all over next day, then finds his throat sore, before Sunday is so hoarse he has to get another to supply his place, gets the "sniffles," and is hawking and hemming and scraping for full two weeks, during which time he is "too poorly" to take any exercise. All violent exercise is a positive injury; all games requiring violent exercise or great exertion, do more harm than good, physiologically; to say nothing of the moral evils, loss of time, temptations to drink, to bet, to swear, to cheat, to lie and to play the fool generally. The exercise which is worth more than all medicine in maintaining health and in curing disease is that which is regular, steady, uniform, deliberate, persisted in day after day, rain or shine. If it rains, take an umbrella and let it rain on; if it is cold, walk or work the faster; if it is windy, turn round and go the other way; if it rains, hails, snows and blows all at once and you have to stay in-doors, then live on bread and water that day, not an atom else, and you will need no exercise to work it up.

